# An In-Vitro Study of the Expansion and Transcriptomics of CD^4+^ and CD^8+^ Naïve and Memory T Cells Stimulated by IL-2, IL-7 and IL-15

**DOI:** 10.3390/cells11101701

**Published:** 2022-05-20

**Authors:** Brooks Hopkins, Justin Fisher, Meiping Chang, Xiaoyan Tang, Zhimei Du, William J. Kelly, Zuyi Huang

**Affiliations:** 1 Department of Chemical and Biological Engineering, Villanova University, PA 19341, USA; bhopki01@villanova.edu (B.H.); jfishe19@villanova.edu (J.F.); 2 Process Cell Sciences, Biologics Process R&D, Merck & Co., Inc., Kenilworth, NJ 07033, USA; meiping.chang@merck.com (M.C.); xiaoyan.tang@merck.com (X.T.); zhimeidu@gmail.com (Z.D.)

**Keywords:** IL-2, IL-7, IL-15, T cell therapy, T cell subsets, RNA-sequencing, fuzzy-model

## Abstract

**Simple Summary:**

T cell-based therapies can be costly, inconsistent, and differentially effective for patients. The limiting reagent for T cell-based immunotherapies is the T cell itself. T cell growth has been linked to endogenously delivered cytokines and messenger proteins that indicate the status of inflammation of the local system in the body. IL-2, IL-7, and IL-15 are the specific cytokines that have been identified in combination to deliver accelerated growth to different T cell subsets. This study focuses on the growth of T cell subsets subject to different combinations of IL-2, IL-7, and IL-15 in the experiment. Based on growth data, mathematical models were developed to project the optimal growth conditions for CD^4+^ and CD^8+^ Naïve and Memory T Cells. RNA sequence data is finally analyzed to study the gene networks that influence growth pathways, specifically for CD^4+^ and CD^8+^ naïve and memory subsets.

**Abstract:**

The growth of T cells ex vivo for the purpose of T cell therapies is a rate-limiting step in the overall process for cancer patients to achieve remission. Growing T cells is a fiscally-, time-, and resource-intensive process. Cytokines have been shown to accelerate the growth of T cells, specifically IL-2, IL-7, and IL-15. Here a design of experiments was conducted to optimize the growth rate of different naïve and memory T cell subsets using combinations of cytokines. Mathematical models were developed to study the impact of IL-2, IL-7, and IL-15 on the growth of T cells. The results show that CD^4+^ and CD^8+^ naïve T cells grew effectively using moderate IL-2 and IL-7 in combination, and IL-7, respectively. CD^4+^ and CD^8+^ memory cells favored moderate IL-2 and IL-15 in combination and moderate IL-7 and IL-15 in combination, respectively. A statistically significant interaction was observed between IL-2 and IL-7 in the growth data of CD^4+^ naïve T cells, while the interaction between IL-7 and IL-15 was found for CD^8+^ naïve T cells. The important genes and related signaling pathways and metabolic reactions were identified from the RNA sequencing data for each of the four subsets stimulated by each of the three cytokines. This systematic investigation lays the groundwork for studying other T cell subsets.

## 1. Introduction

One focus in the current evolution of T cell therapy is concerned with the aspect of ex vivo growth due to the risk of losing batches of patient T cells because of inadequate cell growth rates. Growing T cells is a costly process to inevitably produce chimeric antigen receptor (CAR)-T cells. Medications like Kymriah, a commercially available CAR-T cell therapy, can cost hundreds of thousands, if not in the millions of dollars, if multiple treatments are required for a patient. One way to improve patient cell viability ex vivo is by creating a robust growth cycle protocol for different T cell subsets. There are many ways that CAR-T cells can be synthesized from effector, naïve, memory, or even regulatory T cell subsets. In certain situations, homogeneous batches of cell subsets can be critical for combating certain types of cancers [1,2]. Some cancers require a more heterogeneous blend of T cell subsets [3]. What is consistent, though, is the need to maximize the expansion of the cells while minimizing cell death. In this study, naïve and memory T cell subsets from CD^4+^ and CD^8+^ cells were the focus because each subset has selective relevance to the adoptive T cell therapy umbrella. For example, CD^8+^ memory T cells have been shown to be critical to patients using CAR-T cell therapy against chronic lymphocytic leukemia [2]. In murine models, it has been shown that a heterogeneous combination of CD^4+^ naïve T cells and CD^8+^ memory T cells seemed to benefit the host most due to the enhanced cytotoxicity of the CD^8+^ memory T cells and the ability to augment cytokine response from CD^4+^ naïve T cells [3]. Naïve T cell subsets, both of CD^4+^ and CD^8+^ seem to be of clinical relevance to pediatric patients specifically, whose prognoses seem to rise with greater naïve T cell populations [1]. Additionally, a recent review suggests that CD^4+^ T cells have greater persistence and viability long-term than their CD^8+^ counterparts [4].

In several foundational literature contributions, CD^4+^ naive and memory T cells responded to IL-7 and IL-15, respectively, at 25 ng/mL concentration, and in combination with dendritic-cell derived cytokines also for selective expansion [5]. In other studies, it was found that IL-15 contributed significantly to the expansion of CD^8+^ naïve and memory T cells [6]. IL-2 has been shown to contribute to improvements in CD^4+^ naïve T cell and recent thymic emigrants (RTE) growth when combined with IL-7, as has been shown in our previous work [7]. IL-2, IL-7, and IL-15 clearly have an impact on the growth of T cell subsets, and it is our objective to further extend our previous work from CD^4+^ naïve T cells to other CD^4+^ and CD^8+^ subsets and understand how to optimize the growth of these T cells by implementing various combinations of IL-2, IL-7, and IL-15. In addition, the mRNA transcripts for each selected subset were studied to discern what genes are acting for favorable cellular conditions, specifically related to rapid growth.

The work conducted in this study is outlined below. For each T cell subset, a growth study was conducted whereby frozen cells, purified by their subset, were exposed to a seven-day growth protocol. During this growth study, the cells were subject to various concentrations of IL-2, IL-7, and IL-15 simultaneously to understand the impact of combinations of cytokines on the growth rate. Using the growth data for each subset, adaptive neuro-Fuzzy inference systems (AN-FIS) were created using machine-learning techniques to optimize the growth rate and predict the impact of the three cytokines on the growth rate. Bearing equal importance is studying the mRNA transcripts from each T cell subset at two different time points during the growth cycle using RNA-Sequencing. Finally, an attempt was made to identify the gene regulatory networks to which the mRNA transcripts are related to suggest future gene targets of exploitation.

## 2. Materials and Methods

### 2.1. Cell Culture, Cytokine Stimulation, Quantification

Isolated cells were obtained from HumanCells Biosciences (Fremont, CA, USA), with each examined subset isolated from peripheral blood mononuclear cells prior to receipt. Naïve cells were identified by CD45RA^+^/CD45RO^−^, and memory cells were identified by CD45RO^+^. Each T-cell subset was subject to the same in vitro conditions. Each cell type was thawed from cryopreservation and seeded onto a 96-well culture plate in X-VIVO 15 medium on Day 0 and supplemented with 6 ng/mL IL-2 along with anti-CD3/anti-CD28 Dynabeads (Gibco, Waltham, MA, USA) at a 1:1 bead to cell ratio. On day 3, the cells were counted via hemocytometer, and cytokine stimulation was performed per the established cytokine conditions (Table 1). Cells were lysed on Day 4 and Day 7 for transcriptomic RNA Sequencing using the Qiagen RNeasy Plus Mini kit (Qiagen, Hilden, Germany), according to the manufacturer's protocol. Finally, between days 3 and 7, cell hand counts were completed for cell growth in triplicate.

### 2.2. Growth Plate

**Figure 1:**
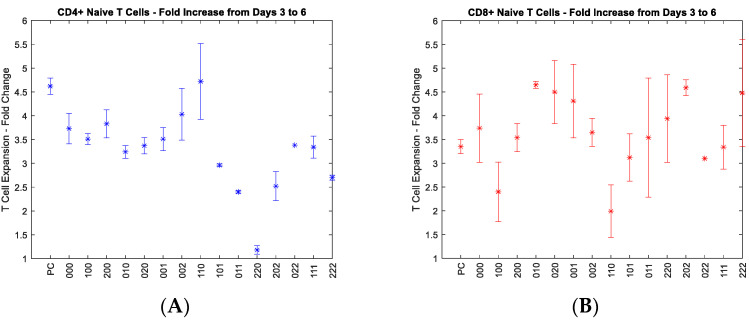

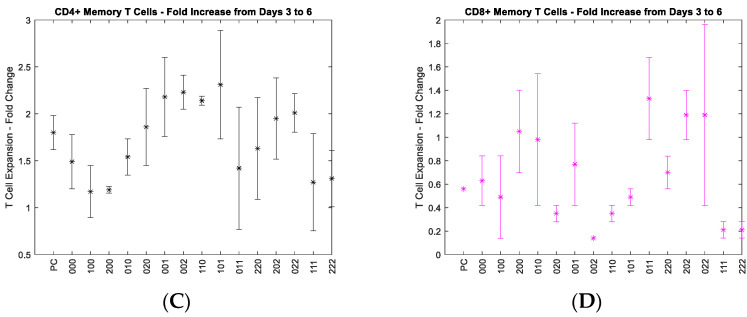
Average (*n* = 3) fold-change plots for all T cell subsets and corresponding conditions: (**A**) naïve CD^4+^ T cells, (**B**) naïve CD^8+^ T cells, (**C**) memory CD^4+^ T cells, (**D**) memory CD^8+^ T cells. The X-axis represents each experimental condition. For example Index 0, 1, and 2 represents the minimum (low), the medium (middle) and maximum (high) level for each cytokine as shown in Table 1, with 0 standing for 10 ng/mL (IL-2), 1.5 ng/mL (IL-7), and 10 ng/mL (IL-15); 1 standing for 33 ng/mL (IL-2), 36 ng/mL (IL-7), 36 ng/mL (IL-15); 2 standing for 60 ng/mL (IL-2), 80 ng/mL (IL-7), and 80 ng/mL (IL-15). The “no cytokines” condition in Table 1 is denoted here by “PC” for the control condition. For example, “010” would represent low IL-2 concentration, moderate IL-7 con-centration, and low IL-15 concentration. These concentrations were selected on the basis of our previous work [14] and literature review.

Instead of using a typical exponential growth equation, the study represents growth as a function of fold change. The fold change, *G*, is defined as the quotient of the ending and beginning cell numbers, as shown in Equation (1):

(1) $$G=\frac{C_{f}}{C_{i}}$$

where $C_{i}$ and $C_{f}$ are live cell concentrations at times $t_{i}$ and $t_{f}$_,_ respectively. The average fold change for each subset was then compared with one another to demonstrate the parity and disparity of the data sets. Below in Table 1, the growth condition is displayed with a key representing the concentrations of each of the selected γ-chain cytokines used. The condition key consigns “0” for being the lowest concentration for each cytokine, “1” being assigned to the medium-level concentration for each cytokine, and “2” being assigned to the highest concentration of each cytokine, in the order of IL-2, IL-7, and IL-15. For example, “200” would represent the highest concentration of IL-2 and the lowest concentrations of IL-7 and IL-15. The “no cytokines” condition in Table 1 is denoted by “PC” in Figure 1.

### 2.3. Transcriptomics Analysis

Six wells each of conditions 000, 200, 020, and 002 (as shown in Table 1) were replicated on the second 96-well plate for RNA extraction at two-time points (days 4 and 7). Four conditions were chosen to analyze, which represent the extreme condition for each of the three cytokines, and a control condition, specifically to understand the impact of each cytokine on the signaling within the cells. Two different time points were chosen on days 4 and 7. Days 4 and 7 were selected for several reasons. With limited resources, it was imperative to demonstrate a notable difference between the time the media was introduced to the cells and the end of the experiment. Day 3 was the point at which the media was introduced, therefore, day 4 was selected to be the day of immediate response. As the experiment carried on to day 7, a final frame of reference was established as growth was saturating due to media exhaustion. At each time point, three wells for each of these conditions were pooled and the cells were lysed with the Qiagen RNeasy Plus Mini kit (Qiagen, Hilden, Germany). The lysates were frozen at −70 °C until RNA isolation could be performed. RNA isolation was performed using the Qiagen RNeasy Plus Mini kit (Qiagen, Hilden, Germany), according to the manufacturers protocol. RNA isolate quality was confirmed prior to shipment for sequencing and was measured using a spectrophotometer (260 nm/280 nm ratio). Sequencing the isolated total RNA was performed by the Center for Medical Genomics, Indianapolis, Indiana. The following protocol was used for RNA seq analysis: Spliced Transcripts Alignment (STAR) software was used to conduct alignment analysis for the FASTQ data [8]. Next, the NGSUTils software suite was used to obtain the initial reads from the RNA Sequencing data [9]. The program *featureCounts* was used for counting reads produced from RNA Sequencing tests [10]. A program named edgeR was used to analyze the gene expression data and perform a number of analytical functions to examine the count data [11]. Finally, based on the expression profiles returned by edgeR, volcano plots were used to identify genes that significantly up/down-regulated for each T cell subset stimulated each of the three cytokines. The genes were then analyzed by the programs Metacore and IPA to delve into process networks for studying the impact the cytokines are having on the pathways that are being activated. In particular, Metacore classifies genes as which contribute to specific pathways, where filtering and reporting on the negative value of the log of the *p*-value, which is below 0.05, with a log2 ratio of greater than 0.5, is completed. In addition, the functions of important genes were studied with a common pathway analysis tool named GeneMania [12] GeneMania uses commonly mapped signaling pathways and employs predictive tools to make connections between how genes might interact or factually how they do interact if there is evidence to support such a claim. Understanding the overlap of these genes is critical to determine if pathways act synergistically, antagonistically, or have no bearing on one another. These genes identified can also serve as potential targets in the future for exploitation.

## 3. Results

### 3.1. Identify Optimal Cytokine Combinations from the Statistical Analysis of Cell Growth Rates

A growth study was conducted for each of the following T cell subsets: CD^4+^ naïve, CD^4+^ memory, CD^8+^ naïve, and CD^8+^ memory T cells. Figure 1 demonstrates the fold change in cell number of each subset. The growth rate data also contains a positive control, denoted by “PC” where cytokines were not added to the media. In “000” condition, cytokines are at their lowest levels to most closely mimic baseline levels [13].

Figure 1A illustrates the growth of the naïve CD^4+^ T cells. Previous work had suggested that the growth rate of naïve CD^4+^ T cells improved through the combination of moderate IL-2 and IL-7 concentrations [14]. A finding was reproduced as IL-2 and IL-7 at moderate concentration return the highest growth rate of any conditions shown [14]. Another finding also shows that the condition independently stimulated by either the highest IL-2 or the highest IL-7 returns strong growth. Our previous work corroborates the finding that a higher concentration of IL-2 independently is associated with greater expansion of these T cells [14]. Other literature contributions can corroborate the importance of IL-7 for survival and this T cell subset entering the cell cycle and replicating as well [15]. The last important finding was the condition of high IL-2 and high IL-7 combined, contributing to a lack of cell growth throughout the study. Previous literature would suggest that fas-mediated apoptosis can be accelerated when using high concentrations of IL-2 while using IL-7 in combination [7]. A strong interaction has been proven statistically in previous work, so this finding is not new, but rather the reproducibility strengthens the scientific conviction and corroboration of this point.

Figure 1B depicts the growth of CD^8+^ naïve T cells. The CD^8+^ naïve T cells demonstrated some intriguing findings during the growth study, in both corroborating previous literature with high IL-15 concentration facilitating expansion, and high IL-7 concentration being of the greatest contributor to growth, independently [6]. CD^8+^ naïve T cells have been subject to studies in the past whereby activation and expansion take place under IL-15 stimulation. Further, Figure 1C illustrates the growth rate of CD^4+^ memory T cells, which appear not to have growing conditions that elicit incremental growth above the average in this study, though all the highest conditions mostly have to deal with IL-7 and IL-15, which again has been corroborated by the likes of Geginat et al. [5]. While in a static environment replete with other lymphocytes, memory T cells are in very low concentration, typically, and often when exposed to antigen-presenting cells (APCs), memory cells do struggle at times with expansion versus apoptosis. Finally, Figure 1D represents the growth rate of CD^8+^ memory T cells, which sustained regression in many conditions. The highest growth by far was elicited by conditions involving IL-15 such as ”011,” ”202,” and ”022”. These conditions eliciting higher growth in CD^8+^ memory T cells should be unsurprising given the previous work contributed by Kanegane and Tosato where IL-15 provides significant improvement of growth [16]. Even though the ”002” condition did lead to a reduced growth rate, when paired with IL-7 or IL-2 independently, there was significant growth achieved.

As discussed above, the data shown in Figure 1 shows the impact of the three selected cytokines on the growth of individual T cell subgroups. The data was further implemented to develop a Fuzzy model to predict the growth rate from the concentrations of the three cytokines. Readers interested in modeling can further refer to Supplementary Material S1 for more detail on it.

### 3.2. Investigate the Interaction of the Three Cytokines in Promoting the Growth of CD^4+^ and CD^8+^ T Cells

For each subset, the statistical significance of the interactions each cytokine had on the growth rate and with each other were explored using the R program presented in our previous work [14]. The approach bears significance in recognizing that cytokines have different impacts on the different T cell subsets, and the interactions can play a role in understanding the likelihood that a particular protocol will impact a batch of T cells in a positive or negative way. Table 2 below records the *p*-value showing the statistical significance of each interaction, with an “X” denoting statistical significance below a *p*-value of 0.05.

CD^4+^ naïve T cells were impacted by IL-2 and IL-7 independently via the growth conditions that clearly had an impact on the growth rate. This is underpinned by the statistical significance shown for IL-2 impacting the growth of CD^4+^ naïve T cells, as well as IL-7. In this study, there was also a statistically significant interaction shown between IL-7 and IL-15, which did not manifest in our previous work. In our previous work, we indicated that CD^4+^ naïve T cells also had a significant interaction between IL-2 and IL-7 [14]. Finally, there was also a significant interaction between all three cytokines. CD^4+^ memory T cells did not produce any statistically significant interactions between the γ-chain cytokines. CD^8+^ naïve T cells also exhibit a statistical interaction between IL-7 and IL-15, as well as between all three cytokines. CD^8+^ memory T cells had statistically significant interactions only between all three cytokines (IL-2, IL-7, and IL-15).

### 3.3. Study Genes Indicating the Purtabation in the Signal Transduction of Activated CD^4+^ and CD^8+^ T Cells Stimulated by Individual Cytokines Using RNA-Sequencing

**Figure 2:**
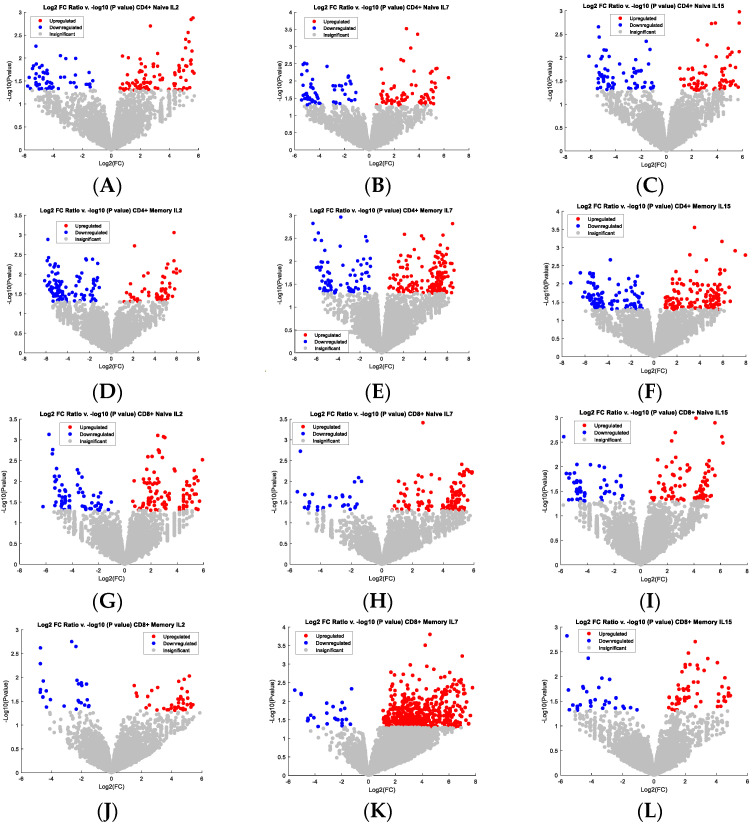
(**A**) CD^4+^ naïve T cells response to highest IL-2 concentration (condition “200”); (**B**) CD^4+^ naive T cells response to highest IL-7 concentration (i.e., condition “020”); (**C**) CD^4+^ naive T cells response to highest IL-15 concentration (i.e., condition “002)”; (**D**) CD^4+^ memory T cell response to highest IL-2 concentration; (**E**) CD^4+^ memory T cell response to highest IL-7 concentration; (**F**) CD^4+^ memory T cell response to highest IL-15 concentration; (**G**) CD^8+^ naïve T cell response to highest IL-2 concentration; (**H**) CD^8+^ naïve T cell response to highest IL-7 concentration; (**I**) CD^8+^ naïve T cell response to highest IL-15 concentration; (**J**) CD^8+^ memory T cell response to highest IL-2 concentration, (**K**) CD^8+^ memory T cell response to highest IL-7 concentration; (**L**) CD^8+^ memory T cell response to highest IL-15 concentration.

During the study, volcano plot analysis was used to show further the proportion of genes that were significantly upregulated or downregulated when being compared to the base condition of “000.” The highest-concentration condition for each cytokine was compared against the base condition for each T cell subset. IL-2, IL-7, and IL-15 have an individual impact on the transcripts within the cell, therefore are a total of 12 volcano plots below (Figure 2) for up-regulated genes (RED, *p*-value lower than 0.05) and down-regulated genes (BLUE, *p*-value less than 0.05) to represent each cytokine and each subset. Though the genes are not called out specifically in Figure 2 due to a large number of those genes, the detail of these genes can be found in Supplementary Material S2. The pathways associated with these genes were further identified with the tools MetaCore and IPA. Principal Component Analysis also validated these genes, which is where the introduction of the RNA-sequencing time points first came into play [17]. Time was the variable to be monitored and was the factor for each of the principal components, so the difference in the fold change of the genes being monitored over two different time points became critical for that purpose. These figures are added in Supplementary Material S3. Genes identified from the volcano plot analysis are available in that Supplementary File. It is also important to note that the genes shown in the PCA plots in the Supplementary Files are also represented visually in the volcano plot analysis. In other words, the same criteria to create the volcano plot analysis were used to create the PCA, i.e., *p*-value and Log2 fold change ratio of plus or minus fifty percent.

The genes with significant up/down-regulation were further analyzed in MetaCore and IPA. The analysis indicates that CD^4+^ naïve T cells are significantly impacted by IL-2, where pathways involving apoptosis, inflammation, metal ion transport, MIF signaling, and neurogenesis appear to be significantly upregulated. A key gene, SOX2, was also upregulated by IL-2, along with ZNF-224, a transcriptional repressor. Tumor Necrosis Factor (TNF) was significantly downregulated. This T cell subset also responds to IL-7, impacting transcriptional regulation, and IL-15 which promotes positive regulation of cell proliferation and signal transduction of the ESR1 nuclear pathway and hedgehog signaling.

CD^8+^ naïve T cells are also significantly impacted by IL-2, impacting pathways involved in neurogenesis, cell adhesion, and inflammation. Additionally, processes involved in cell fate, particularly Notch signaling, were impacted. The upregulation of IL-5 and CNK2 was observed. The MYC pathway, SOX2, and RORC were also downregulated by IL-2. A plethora of genes were downregulated by IL-7 in the CD^8+^ naïve T cells, including STAT5B, FOXP3, and MYC, all involved in regulating cell proliferation. The data suggest that IL-7 impacts the CD^8+^ naïve T cells by impacting signaling pathways involved in Th-17 helper cells derived cytokine inflammation, blood vessel morphogenesis, TGF-β, GDF and interferon signaling, and finally, bone remodeling. IL-15 impacts nerve impulse transmission, and ECM remodeling, in addition to TREM-1 and chemotaxis signaling.

**Figure 3:**
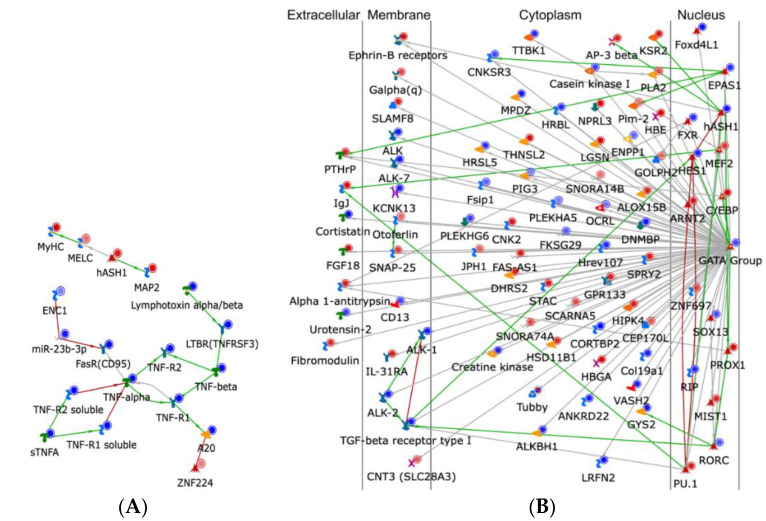
Example pathways regulated by IL-2 in CD^4+^ naïve T cells (**A**) and CD^8+^ naïve T cells (**B**). These figures were generated in the software Metacore. The meaning of these icons can be found in Metacore. Further explanation of the key legend is explained in Supplementary Material S6.

It is also important to observe CD^4+^ and CD^8+^ naïve T cell subsets to draw parity in their tendency to respond to IL-2. When responding to this cytokine, pathway upregulation ensues for hedgehog signaling, neutrophil activation, and cAMP response element modulator (CREM) signaling pathway. The CREM signaling pathway is involved in the male reproductive process, impacting spermatogenesis [18]. IL-7 generates activity in the β-catenin, Notch, IP3, VEGF, and Wnt signaling pathways, in addition to synaptogenesis and cardiac development. Finally, IL-15 impacts both CD^4+^ and CD^8+^ naïve T cells by inducing pathways involved in neurogenesis, blood vessel morphogenesis, the JAK-STAT pathway, and positive regulation of cell proliferation. Due to the space constraints, pathways regulated by IL-2 in CD^4+^ naïve T cells and CD^8+^ naïve T cells were shown as examples in Figure 3A and 3B, respectively.

In the memory cell subsets, the cytokines appear to have less impact on genes involved in specific signaling pathways based on RNA sequence data analysis. IL-2 impacts CD^4+^ memory T cells with respect to pathways involved in blood coagulation and antioxidant activity, specifically regarding reactive oxygen species. IL-7 impacts neurogenesis and synaptogenesis pathways. Additionally, IL-7 upregulates NONO and IRF7. Finally, IL-15 impacts pathways involving cell adhesion, manganese transport, and muscle contraction. CD^8+^ memory T cells are impacted by IL-2 with respect to taste-signaling and MAPK activity. Data suggests that IL-7 upregulates FGFR, CD70, and IL6R, as well as pathways involved in muscle contraction, muscle development, cell adhesion, and calcium transport. CD^8+^ memory T cells are impacted by IL-15 with respect to pathways involved in iron transport, Wnt signaling, and phagocytosis. CD^4+^ and CD^8+^ memory T cells have overlapping gene pathways that are differentially expressed, including sodium transport when responding to IL-2. IL-7 impacts both CD^4+^ and CD^8+^ memory subsets by upregulating neurogenesis, phagocytosis, and sodium and calcium transport. Finally, CD^4+^ and CD^8+^ memory subsets impact pathways involved in blood coagulation.

**Figure 4:**
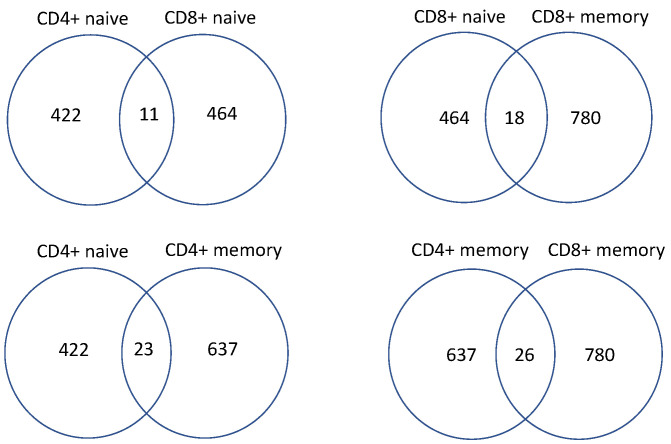
Overlapping views of shared genes that were significantly up/down-regulated by cytokines for different T cell subsets.

Finally, it is important to show examples of overlapping genes between subsets: Figure 4 demonstrates the overlap of significantly-regulated genes between CD^4+^ T cells and CD^8+^ naïve T cells. The detail of the genes shared by different T cell subgroups shown in Figure 4 are provided in Supplementary Material S5. CD^4+^ naïve T cells shared 11 genes with CD^8+^ naïve T cells as differentially expressed, meeting the threshold specified earlier in the results section. Eight of the shared 11 genes were upregulated by IL-7 in both CD^4+^ naïve and CD^8+^ naïve T cells, while two genes were upregulated by IL-15 and one gene was downregulated by IL-15. CD^8+^ naïve and CD^8+^ memory T cells shared 18 genes, among which were 15 genes upregulated by IL-7, one gene upregulated by IL-2, one gene downregulated by IL-7, and one gene downregulated by IL-15. CD^4+^ naïve and CD^4+^ memory shared 23 genes: 13 genes upregulated by IL-7, two genes upregulated by IL-2, one gene down-regulated by IL-7, three genes upregulated by IL-15, and four genes downregulated by IL-15. The greatest number of overlapping genes was seen between CD^4+^ memory and CD^8+^ memory T cells with 26 shared genes, among which were 21 genes upregulated by IL-7, two genes upregulated by IL-2, one gene downregulated by IL-7, one gene upregulated by IL-15, and one gene downregulated by IL-15.

## 4. Discussion

### 4.1. The Impact of Each Cytokine on T Cell Growth

#### 4.1.1. Use of Fold Change over Growth Rate

In a typical growth study, the growth rate is calculated via a logarithmic function consistent with determining the exponential growth. The equation is represented by taking the quotient of the natural logarithm of the ratio of the cell numbers at Time Two and Time One, respectively, and the difference between the two-time points. In this study, one focus was on the fold change of the T cell, meaning the quotient of the final cell number and the initial cell number only. Fold change was used in lieu of the growth rate due to the nature of the differences in patterns between the T cell subsets. Fold change better represents and conveys the details behind how the cells grew for each condition, rather than expressing the rate of growth or the difference in cell concentration, both of which appear more arbitrary in this case.

#### 4.1.2. Impact of Cytokines on the Growth of CD^4+^ and CD^8+^ T Cells

Each T cell subset appeared to be impacted by specific cytokines and combinations therein, which were distinct from one another. Reflecting on our previous work and comparing to the current results, CD^4+^ naïve T cells, in both circumstances, reacted strongly to the condition key “110,” which consigns moderate IL-2 and IL-7 concentrations, respectively. Previously mentioned was the fact that IL-2 and IL-7 had a statistically significant interaction, one which had been previously mentioned in literature [7]. The combination of these two cytokines, specifically at high concentrations, has been described to contribute to Fas-mediated apoptosis, being that IL-2 can antagonize IL-7 at higher concentrations. Though, IL-2 appears to synergize at lower concentrations. Further evidence to support this theory is the significantly lower growth rate of the CD^4+^ naïve T cells when under stimulation of higher concentrations of IL-2 and IL-7 in combination, reflected by key “220”. As IL-2 and IL-7 are shown to have a statistically significant impact on the growth process independently, it should be unsurprising that these conditions return higher growth rates relative to the other conditions executed.

With respect to CD^8+^ naïve T cells, the highest growth rate was achieved with the condition of moderate IL-7 concentration, with key “010,” which was relatively unexpected, though there is evidence to support such a claim in murine CD^8+^ T cells [19,20]. High IL-7, with key denoted by “020,” was also shown to return a high growth rate. The lowest growth rate relative to all other conditions was associated with key “110,” which is consigned by moderate IL-2 and moderate IL-7.

CD^4+^ memory T cells showed little correlations between growth conditions, though the highest conditions in producing growth response were associated with moderate and high IL-15, as well as moderate IL-2 and IL-15 in combination. CD^8+^ memory T cells responded least to the condition “002” (i.e., high IL-15 concentration), where the highest condition was “011” (i.e., moderate IL-7 and moderate IL-15). Unfortunately, CD^8+^ memory T cells experienced regression in growth for many of the different conditions to which they were exposed. This may be due to a discrepancy between the autocrine, paracrine, and endocrine signaling tendencies of these cells. A larger range of cytokine concentrations may be needed to explore CD^8+^ memory T cells in future research.

### 4.2. Discussion of Genes Regulated by Cytokines in CD^4+^ and CD^8+^ T Cells

When strictly observing CD^4+^ naïve T cells, the gene analysis revealed that some of the major pathways differentially expressed when stimulated by IL-2 included those relating to macrophage migration inhibitory factor (MIF) signaling, a protective effect against infection, and other potential damage to organs via autocrine and paracrine signaling [21,22]. In our previous work, we stated that many of the genes differentially expressed by stimulation from IL-2 also were involved in anti-microbial defenses and inflammation. Here some of those results were reproduced. Many genes regulated by the stimulation of IL-15 were involved as positive indicators of cell proliferation, hedgehog signaling, and ESR1 signal transduction. Hedgehog signaling, although not completely understood, refers to the pathway that acts as a directing agent for nascent cells, which acts through many steps of signal transduction [23]. The ESR1 gene refers to estrogen receptor signaling, which is involved in the progression of breast and endometrial cancers in female subjects [24].

When observing CD^8+^ naïve T cells, the pathways that were upregulated via IL-2 include Notch signaling, IL-5 signaling, leptin signaling, cell adhesion, and neurogenesis. Notch signaling refers to an evolutionarily ancient cell interaction mechanism in which cell fates are dictated by the signals exchanged between neighboring cells through the Notch receptor [25]. IL-5 signaling deals with the regulation of eosinophils, specifically differentiation [26]. Unlike CD^4+^ naïve T cells, the software program IPA showed that CD^8+^ naïve T cells were impacted by IL-2 downregulating SOX2. This gene is involved in cell fate processes. IL-7 has a particular correlation to differentially express genes that make up pathways involved in cell adhesion and T helper cells-17 (Th-17) derived cytokine inflammation. IL-15 regulates genes involved in extracellular matrix (ECM) remodeling and TREM-1 signaling. ECM remodeling occurs throughout the life of the T cell due to aging, and when cross-linking of ECM from different T cells occurs, loss of mobility can occur, thus sending the cross-linked cells down a path that would eventually lead to their demise [27]. Thus, ECM remodeling can be valuable to ensure cells maintain their individuality. TREM-1 signaling refers to the activation receptor that causes inflammation, cytokine synthesis, and the stimulation of neutrophils [28].

As for CD^4+^ memory T cells, IL-2 influenced antioxidant activity and molecular carrier activity, along with the hydrogen peroxide metabolic process. These gene functions all relate to one another insomuch as they all denote aerobic metabolism in a cell. Reactive Oxygen Species (ROS) can relate to antioxidant activity, i.e., the cell attempting to protect itself from oxidative stress, and the hydrogen peroxide metabolic process can be an output of heavy aerobic metabolism. IL-7 upregulates NONO and IRF7, genes involved in CREB signaling and regulation of immunity against viruses, respectively [29,30]. IL-15 plays a role in stimulating genes which are parts of pathways involved in muscle contraction, cell adhesion, and manganese transport.

When observing the CD^8+^ memory T cells, IL-2 appears to have had more of an impact on MAP-kinase activity and the extracellular matrix organization. From a pathway perspective, the MAP-kinase piece is rooted in the regulation of cellular responses to cytokines. As mentioned in the results section, FGFR and CD70 were differentially expressed and are tied to the growth and activation of T cells. On the other hand, IL-15 impacts genes involved in pathways that regulate iron transport, Wnt signaling, and immune response related to phagocytosis. Wnt signaling is involved in the direction of cell differentiation and embryonic development; therefore, IL-15 plays a role in the downstream cell fate [31]. Phagocytosis is the process by which specific immune cells trigger a defense mechanism to degrade and eliminate APC threats by engulfing the threat, subsequently using destructive chemical compounds to eradicate the threat.

The gene analysis reveals parity between the CD^4+^ and CD^8+^ naïve cells and between the CD^4+^ and CD^8+^ memory T cells. For the naïve subsets, IL-2 significantly changed the expression of genes involved in hedgehog signaling, which is important in paracrine signaling style between cells to consolidate messages and gain tacit compliance and uniformity of expression. Additionally, IL-2 significantly increased inflammation in both subsets. This should not come as novel but rather as a confirmation of many previously known studies, where IL-2 causes inflammatory and apoptotic-related pathways to become upregulated. Finally, the CREM signaling pathways have also become upregulated. IL-7 plays a key role in impacting differentially expressed genes involved in β-catenin, Notch, IP3, Wnt, and VEGF signaling. These pathways are all involved in paracrine signaling between cells to consolidating early-phase growth signals, differentiation, and progression of cell fates. Genes shared by the stimulation of IL-15 include those associated with positive regulation of proliferation, JAK-STAT pathway regulation, and blood vessel morphogenesis. The JAK-STAT pathway is one directly related to the growth of the cell.

For the memory subsets between CD^4+^ and CD^8+^ memory T cells, IL-2 played a very insignificant role in delivering any significant and consistent results of parity between the CD^4+^ and CD^8+^ memory T cells. Though, IL-7 played a significant role in upregulating genes involved in calcium and sodium transport and neurogenesis. Finally, IL-15 impacted genes involved in blood coagulation. Genes that have been commonly up- or down-regulated between different subsets are located in Supplementary Material S5 for further information.

Finally, it is important to highlight the overlapping genes between subsets of T cells shown in Figure 4. Beginning with the overlap of CD^4+^ and CD^8+^ naïve T cells, they collectively share 11 genes. They do not share any genes when stimulated by IL-2, which have been either up or down-regulated. IL-7 plays a greater part, including most of the genes that overlap between the two subsets. CMPK2, which is a kinase involved in nucleotide synthesis, is shared via upregulation by stimulation from IL-7. Other genes upregulated by IL-7 shared include PARP9, which is involved in defense functions and response to IFN-𝛾; RSAD2, which is involved in response to interferons; ERC2, which is involved in neurotransmitter regulation; and ADRA2C, which is involved in c-AMP signaling and activation of protein kinase activity. All of these genes seem to be involved in increased cellular activity and inflammation; with response to interferons, neurotransmitter regulation, and nucleotide synthesis, IL-7 appears to be upregulated genes in both CD^4+^ and CD^8+^ naïve T cells as a result of its affinity to enhance survival and aid in proliferation, as evidenced in the growth experiments.

When observing the comparison of CD^8+^ naïve and CD^8+^ memory T cells overlapping genes, we see an upward trend of 18 overlapping genes. Again, they are nearly all driven by upregulation by IL-7. Genes such as CD1C, which is a transmembrane protein involved in the antigenic presentation to antigen-presenting cells (APCs); SALL4, which is involved in stem-cell population maintenance; JCHAIN, which is involved in tissue homeostasis and ADGRV1, which is involved in neuronal pathways. The trend of several of these genes relates to homeostasis and conservation of key infrastructure. The rest of the genes are non-coding regions whose functions are not yet known.

The greatest number of genes shared was between CD^4+^ and CD^8+^ memory T cells, sharing 26 genes. Genes shared via stimulation of IL-2 are non-coding genes that are unimportant, but again genes upregulated by IL-7 are shared between the naïve and memory T cells the most. For example, BST1 mediates pre-B cell growth; KCNH8 is a potassium voltage gate gene; other genes include PTPRN, which is involved in cell growth, RNASE2, which facilitates anti-microbial activity; and MIR571, which is a micro-RNA without confirmed targets. These genes also seem to be involved in both increased cellular activity, growth, and cell protection.

Genes shared between CD^4+^ naïve and memory T cells number 23. Genes upregulated via IL-2 are small nucleolar and non-coding genes that are largely unimportant, but again genes upregulated by IL-7 were shared the most. CMPK2 was seen again in this list, along with RSAD2. DUBR is involved in transcription, as well as RXRB. Finally, MIR5585 and MIR6840 are two micro-RNAs whose functions are not known, but prediction targets are available open-source. IL-15 also downregulates these micro-RNAs, so there is the potential use of these cytokines in tandem to help remove these genes as silencing mechanisms. Many of these genes are involved in transcription and cellular activity as well.

Overall, IL-7 appears to have had the most dramatic effect on upregulating genes, especially those shared across the T cell subsets we experimented with. IL-7 has consistently, both in literature and experimentation, played a part in both growth and enhanced cellular activity, as seen here with the genes which have been differentially expressed, as seen and shared above. IL-7 has also historically been a potent survival cytokine for T cells. Many of the genes upregulated by IL-7 can be future potential targets, where potential substrates feeding the upregulated genes could be further exploited to enhance growth further without cellular exhaustion of media.

### 4.3. Functionality Assessment

The importance of a follow-up assessment after the growth cycle is completed cannot be understated. It is critical for the clinician or the scientist to ultimately have the confidence that the population of cells is accurately described. After the growth studies were completed, the naïve T cell populations were monitored for differentiation and the preliminary result was obtained (not shown). Using surface markers as a monitoring mechanism carried out using FACS analysis, the CD^4+^ naïve T cells had the lowest shift to memory cells with no cytokines added, i.e., the “PC” condition. The next lowest rate of differentiation was achieved via the “002” condition, or high IL-15, low IL-2, and IL-7. Again, the lowest rate of differentiation in CD^8+^ naïve T cells was also achieved via the “002” condition. The highest rates of differentiation were achieved in CD^4+^ naïve T cells using the highest concentrations of all cytokines in combination, condition “222” and for CD^8+^ naïve T cells, the highest rate of differentiation was achieved via conditions “020” and “022,” which implies that there is an impact on differentiation using IL-7 in CD^8+^ naïve T cells. The “020” condition also nearly returned the highest growth rate in CD^8+^ naïve T cells.

In the memory T cells, a memory-specific shift to central memory cells in CD^4+^ memory T cells occurred most under condition, “200” and in CD^8+^ memory T cells, the highest shift to central memory T cell occurred using condition, “222.” The shift occurred between effector memory and central memory as verified using the surface markers identified using FACS analysis. A consistent trend is seen across naïve and memory T cell subsets, which is illustrated by higher concentrations of cytokines eliciting higher rates of differentiation. Creating the optimal concentration of cytokines whereby the highest rates of growth are achieved with the lowest rates of differentiation is the goal. For all subsets, IL-15 appears to hold the key to low levels of differentiation, with IL-7 appearing to contribute to growth, as seen in the CD^4+^ and CD^8+^ naïve T cells. IL-2 is most effective at expanding the CD^4+^ naïve T cells and favoring differentiation in most all other subsets.

### 4.4. Limitation and Future Work

The naïve T cells were purified by HumanCells Biosciences before the experiment. Once the cells were delivered and transferred to the new environment that was supplemented with anti-CD3/CD28 dynabeads, the naïve T cells may have begun to start differentiating themselves. It is challenging to maintain un-differentiated naïve T cells while expanding the population. Therefore, the results presented in this work are mainly for activated naïve T cells. This work aims to study the optimal cytokine combination for in vitro expansion for this manuscript. This paper is thus focused on differences in the growth and transcriptomics of the four T cell subsets due to cytokine supplementation. While the optimal cytokine combination conditions have been identified in this work, they should be further evaluated in cancer models before clinical application. This is an important research topic for future investigation. Preliminary Fuzzy models were developed in this work to predict the growth of the four T cell subsets stimulated by various combinations of cytokines IL-2, IL-7, and IL-15. To improve the clinical value of these models, more experimental data will be needed further to test the prediction capability of the developed models.

## 5. Conclusions

The growth responses of four different T-cell subsets in a seven-day growth experiment using γ-chain cytokines IL-2, IL-7, and IL-15 were investigated in this work. Overall, the growth rates of the naïve subsets in both CD^4+^ and CD^8+^ were the most responsive to the growth conditions. Moderate IL-2 and moderate IL-7 in combination appears to be the most favorable condition. For CD^8+^ naïve T cells, growth conditions favored moderate and high concentrations of IL-7. CD^4+^ memory T cells responded neutrally to the prescribed cytokine cocktail, lightly favoring conditions of moderate and high concentrations of IL-15, as well as a combination of moderate IL-2 and IL-15 concentrations. While regression was observed in the growth of CD^8+^ memory T cells in most conditions, there were some conditions that performed more efficiently than others, such as moderate and high concentrations of combinations using IL-7 and IL-15. There were significant interactions identified between IL-2 and IL-7 for stimulating the growth of CD^4+^ naive T cells, while the interaction between IL-7 and IL-15 was observed for CD^8+^ naive T cells. Fuzzy models were developed to predict the growth rate of the four selected T cell subsets. The major signaling pathways were identified from the RNA-sequencing data for the four subsets stimulated by each of the three cytokines. IL-2 significantly increased inflammation genes and changed the expression of genes involved in hedgehog signaling for CD^4+^ and CD^8+^ naïve cells. This multiple subset study lays the groundwork to compare other subsets, such as regulatory T cells and effector cells.

## Figures and Tables

**Table 1:** The keys and concentrations of IL-2, IL-7, and IL-15 were used in the experiments.

Condition	Key	IL-2 (ng/mL)	IL-7 (ng/mL)	IL-15 (ng/mL)
1	no cytokines	0	0	0
2	000	10	1.5	10
3	100	33	1.5	10
4	200	60	1.5	10
5	010	10	36	10
6	020	10	80	10
7	001	10	1.5	36
8	002	10	1.5	80
9	110	33	36	10
10	101	33	1.5	36
11	011	10	36	36
12	220	60	80	10
13	202	60	1.5	80
14	022	10	80	80
15	111	33	36	36
16	222	60	80	80

**Table 2:** *p*-values of statistical significance for a stimulus on each T cell subset.

Stimulus	CD^4+^ Naïve	*p* Value	CD^4+^ Memory	*p* Value	CD^8+^ Naïve	*p* Value	CD^8+^ Memory	*p* Value
IL-2	X	0.000457		0.231		0.2338		0.9533
IL-7	X	0.003395		0.828		0.5216		0.6237
IL-15		0.829209		0.221		0.9788		0.4289
IL-2/IL-7		0.076981		0.944		0.1731		0.0654
IL-2/IL-15		0.347061		0.772		0.8352		0.7763
IL-7/IL-15	X	0.008359		0.196	X	0.0141		0.6194
IL-2/IL-7/IL-15	X	0.012759		0.444	X	0.0186	X	0.0126

## Data Availability

Please reach out to corresponding author for additional data.

## Supplementary Materials

The following supporting information can be downloaded at: https://www.mdpi.com/article/10.3390/cells11101701/s1, S1. Fuzzy models for the growth of the four T cell subgroups; S2. the genes significantly up/down-regulated by cytokines IL-2, IL-7, and IL-15 in each of the four T cell subsets that include CD^4+^ and CD^8+^ naïve and memory T cells; S3. Principal Component Analysis of each T cell subset; S4. Figure Legend for the Metacore network; S5. Shared genes up/down-regulated for all T cell subgroups; and S6. Preliminary functionality data of naive T cells.
