# Supplementary material for: An In-Vitro Study of the Expansion and Transcriptomics of CD4+ and CD8+ Naïve and Memory T Cells Stimulated by IL-2, IL-7 and IL-15
Source: Cells. 2022 May 20;11(10):1701. doi: 10.3390/cells11101701 (PMC9139303; doi:10.3390/cells11101701)
Supplement: Supplementary file 1 [file cells-11-01701-s001.zip › Supplementary Material S1_Fuzzy Models Supplemental Materials.pdf]

Supplementary Materials: Fuzzy Modeling

2.4. Development of Fuzzy models for growth dynamics

In this contribution, Fuzzy models were created on the basis of the growth study data for each subset. The models were developed to predict the growth as the quotient of ending and starting cell number of the T cell subsets stimulated by IL-2, IL-7 and IL-15.

For the growth study model, the value of each of the inputs (i.e., concentrations of the γ-chain cytokines) and the output (i.e., the growth rate of T cell subsets) was categorized into a tertiary set of decisions (to reduce the number of model parameters). The growth data was then utilized to generate the Adaptive Neuro Fuzzy Inference System to build the linguistic rules to link the inputs and output. Then Fuzzy model then infers the value of the output on the basis of the variables' membership functions and linguistic rules. The tertiary set of membership functions for each input was kept consistent from the growth model. Each condition was used containing three levels, one for each cytokine concentration. The output was the final proportion of memory T cells, since all conditions began with the same proportion of naïve and memory cell markers. Figure 1 shows an illustrative diagram of the Fuzzy model.

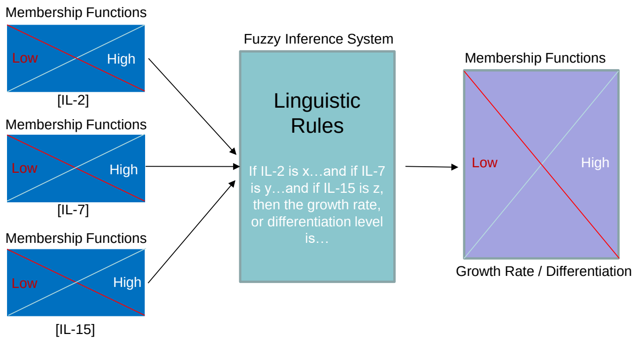

Figure S1. An illustrative diagram of the Fuzzy model to link the cytokine concentrations to the T cell growth rate.

On the basis of the growth data, the Fuzzy model was implemented in the Mamdani format by using the MATLAB command *tunefis*, whereby the Fuzzy Inference System was developed using the bounds of the experiment for each of the cytokines' concentrations. In this instance, the bounds for the cytokine concentrations were 70 ng/mL, 100 ng/mL and 100 ng/mL for IL-2, IL-7 and IL-15, respectively. After the Fuzzy Inference System (FIS) was created and the membership functions were established, the parameters in variables' membership functions were tuned to minimizes the root-mean squared error between the experimental data and the model predicted values. A global minimum solution was obtained by using the particle swarm algorithm followed by a pattern search algorithm. After the rules were established and membership functions were estimated, the Fuzzy model became capable of evaluating future growth conditions as the new inputs.

Results:

Develop a Fuzzy model to predict the growth rates of activated CD4+ and CD8+ T cells for various cytokine combinations

Four adaptive neuro Fuzzy inference systems (ANFIS) were produced using machine learning techniques to model and predict the growth rate of different cytokine combinations for each subset. In Figure 3A, a close fit of the predicted ANFIS model to the experimental data in CD4+ naïve T cells can be demonstrated. The CD8+ naïve T cell data set presented in Figure 3B presented some challenges for Fuzzy modeling, though the final fit was remarkably close. Most of the experimental data points match well with the predicted values. Modeling results for the memory subsets demonstrated weaker correlation.

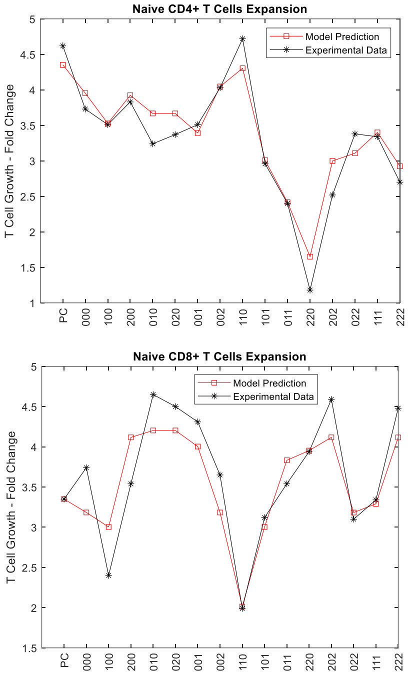

(A) (B)

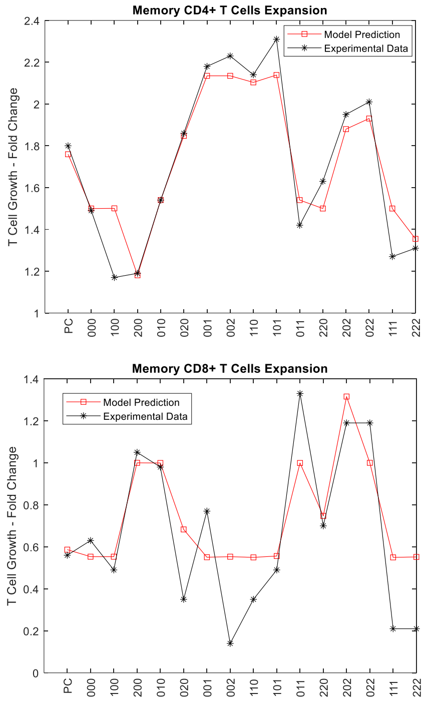

(C)

(D)

Figure S6. Growth plots of naïve and memory CD4+ and CD8+ T cell versus ANFIS model prediction

One key finding here is the ability to use these models to predict future results in follow-up experimental trials of these subsets of T cells. In our previous work, we provided a 3-D surface heat map of optimized treatment zones for the combination of cytokines. Here, we maintain the capability to provide this 3-D surface heat map for each T cell subset, but we provide the 2-D overlay as results in the supplementary materials section because we are not able to fully validate the model with the limited data. We include these models to allow for further work to be carried out using these T cell subsets with the models as the basis for the prediction of future experiments.
