# Supplementary material for: An In-Vitro Study of the Expansion and Transcriptomics of CD4+ and CD8+ Naïve and Memory T Cells Stimulated by IL-2, IL-7 and IL-15
Source: Cells. 2022 May 20;11(10):1701. doi: 10.3390/cells11101701 (PMC9139303; doi:10.3390/cells11101701)
Supplement: Supplementary file 1 [file cells-11-01701-s001.zip › Supplementary Material S3_Principal Component Analysis of each T cell subset.pdf]

Principal Component Analysis of each T cell subset stimulated with each cytokine:

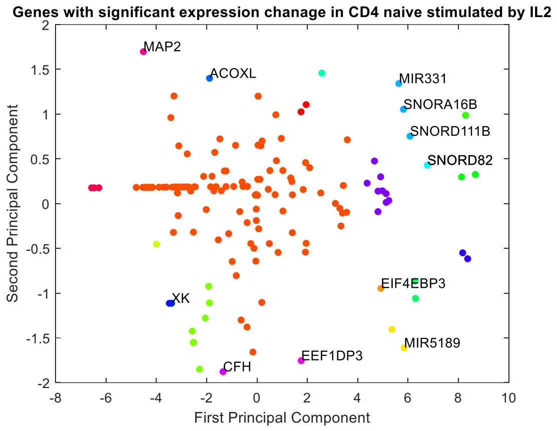

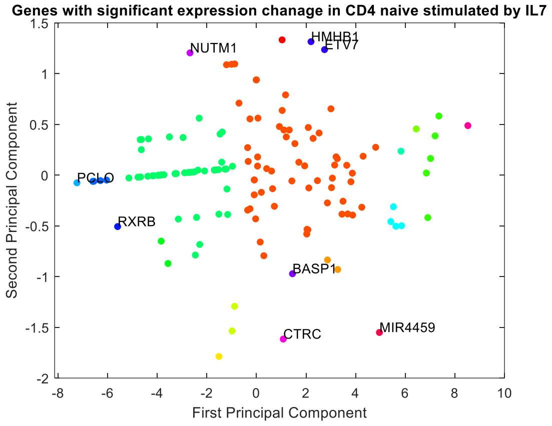

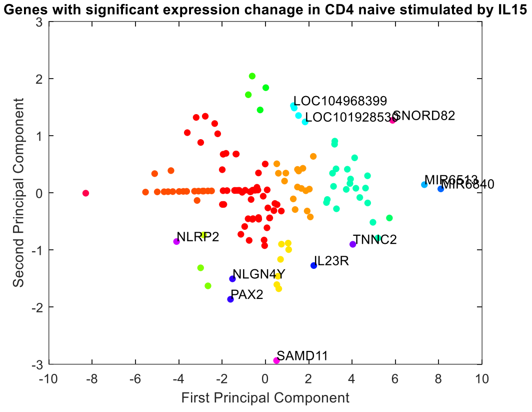

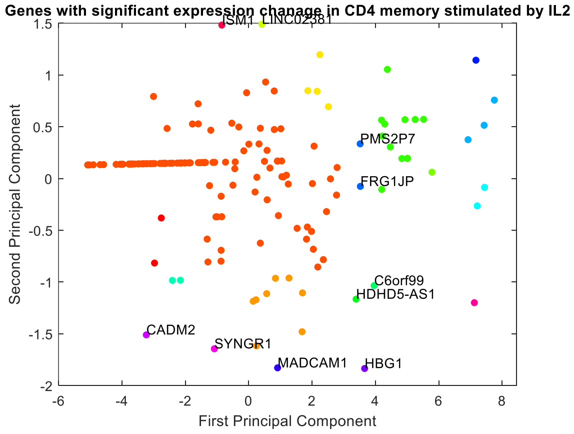

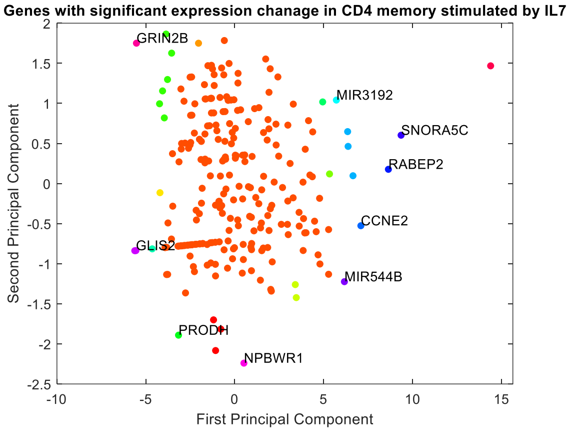

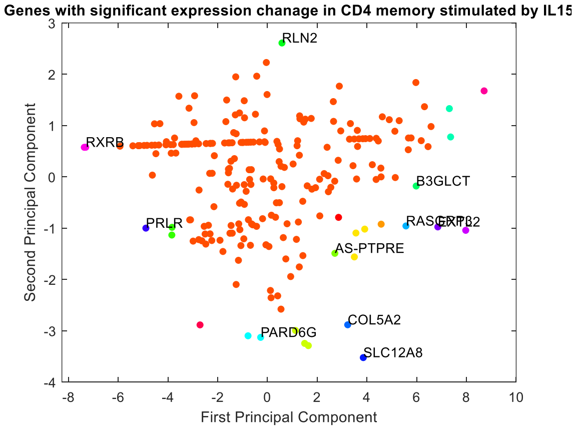

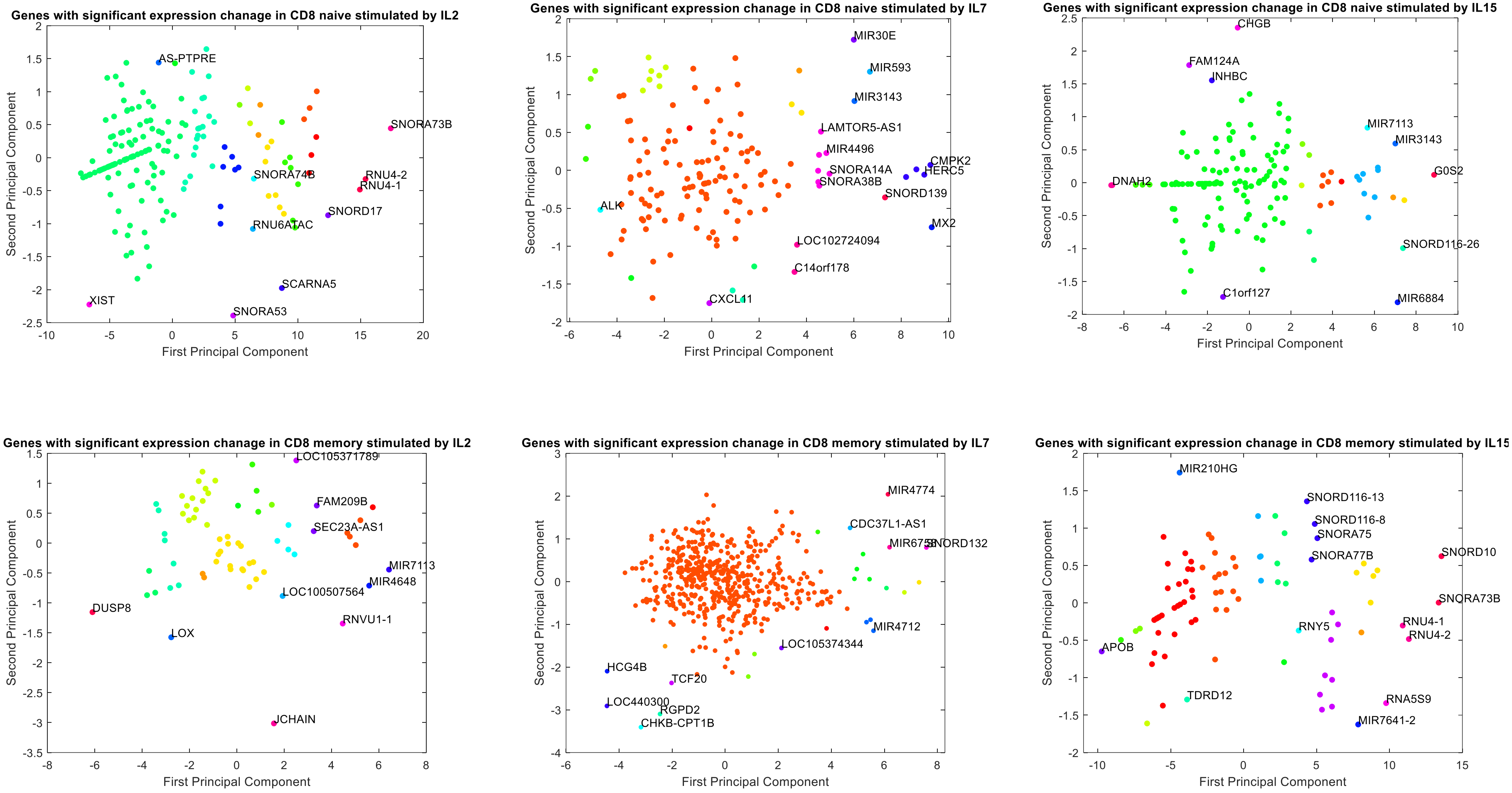

Supplemental Material S3 Figure: The colored points representing genes have been projected into a 2-dimensional space using principal component analysis. The color of the dots represents the distinct cluster of genes as represented by MATLAB. Genes which fall into specific clusters may bear significance to either shared function, co-localization, co-expression, antagonism, or some other type of relationship which links them together. In some of the plots, some clusters contain more genes than others. Again, this can be also because when observing the data through the planes of the first or second principal components, more of the variation may be

able to be explained by the first or second principal component, in which case a cluster may become more pronounced based on the plane drawn through the data. This also falls in line with the variation or lack thereof when concerning what genes fall into what clusters.
