# Supplementary material for: An In-Vitro Study of the Expansion and Transcriptomics of CD4+ and CD8+ Naïve and Memory T Cells Stimulated by IL-2, IL-7 and IL-15
Source: Cells. 2022 May 20;11(10):1701. doi: 10.3390/cells11101701 (PMC9139303; doi:10.3390/cells11101701)
Supplement: Supplementary file 1 [file cells-11-01701-s001.zip › Supplementary Material S5_Functionality of naive T cells.pdf]

Supplementary Material S5:

Table of CD4+ Naïve (CD4+/CD45RA+) T cell shift to Memory (CD4+/CD45RA-) Subset. Columns represent the percentage of cells in each category (naïve v. general memory subsets). Balance of each column is effector subset.

	Day 3 (%)		Day 4 (%)		Day 7 (%)	
Condition	CD4+/CD45RA+	CD4+/CD45RA-	CD4+/CD45RA+	CD4+/CD45RA-	CD4+/CD45RA+	CD4+/CD45RA-
No Cyt	88.87	10.59	78.01	19.7	71.4	28.2
000	88.87	10.59	74.57	23.86	48.31	51.23
100	88.87	10.59	74.16	24.05	49.81	49.8
200	88.87	10.59	74.01	24.03	48.66	51.04
010	88.87	10.59	75.25	23.71	42.05	57.92
020	88.87	10.59	74.67	24.47	53.31	46.23
001	88.87	10.59	67.83	30	48.97	50.53
002	88.87	10.59	75.89	22.67	56.07	43.6
110	88.87	10.59	76.08	23.58	53.44	46.18
101	88.87	10.59	70.48	28.38	41.74	58
011	88.87	10.59	66.44	32.74	44.51	55.19
220	88.87	10.59	66.7	30.77	41.13	58.41
202	88.87	10.59	68.49	29.88	41.34	57.66
022	88.87	10.59	61.42	37.51	38.11	61.41
111	88.87	10.59	66.98	30.95	38.99	60.47
222	88.87	10.59	72.56	25.83	38.26	61.72

Table of CD8+ Naïve (CD8+/CD45RA+) T cell shift to Memory (CD8+/CD45RA-) Subset. Columns represent the percentage of cells in each category (naïve v. general memory subsets). Balance of each column is effector subset.

	Day 3 (%)		Day 6 (%)		Day 7 (%)	
Condition	CD8+/CD45RA+	CD8+/CD45RA-	CD8+/CD45RA+	CD8+/CD45RA-	CD8+/CD45RA+	CD8+/CD45RA-
No Cyt	97.07	2.93	88.45	9.57	82.15	15.77
000	97.07	2.93	86.58	10.48	76.77	19.73
100	97.07	2.93	90.45	9.39	80.13	16.32
200	97.07	2.93	91.65	8.1	79	16.59
010	97.07	2.93	91.75	8.17	81.05	15.56
020	97.07	2.93	89.65	10.25	77.81	18.71
001	97.07	2.93	86.31	12.92	78.85	18
002	97.07	2.93	88.55	11.04	84.82	12.7
110	97.07	2.93	84.06	14.45	82.17	14.8
101	97.07	2.93	82.44	15.6	81.51	15.12
011	97.07	2.93	81.51	15.54	79.63	17.72
220	97.07	2.93	85.57	14.36	77.94	17.87
202	97.07	2.93	84.99	13.38	80.26	17.76
022	97.07	2.93	82.48	14.94	78.36	18.64
111	97.07	2.93	83.23	16.04	79.26	16.47
222	97.07	2.93	85.54	13.16	78.99	17.45
