# Supplementary material for: An In-Vitro Study of the Expansion and Transcriptomics of CD4+ and CD8+ Naïve and Memory T Cells Stimulated by IL-2, IL-7 and IL-15
Source: Cells. 2022 May 20;11(10):1701. doi: 10.3390/cells11101701 (PMC9139303; doi:10.3390/cells11101701)

Supplementary Materials S6

Figure Legend for the Metacore network

Protein interaction networks were built in Metacore to identify the islands of directly interconnected proteins from the differentially expressed gene (DEG) lists. DEG was defined as genes that were up or down regulated with absolute log2FoldChange > 0.5 and p-value < 0.05 in cells treated with IL2 compared to controls. Left panel: network of DEG (comparing IL2-treated to controls) in CD4 naïve cells. Right panel: network of DEG (comparing IL2-treated to controls) in CD8 naïve cells, the network lay out is based on the protein's intracellular localization and some proteins in the network were omitted for clarity. The different protein classes such as transcription factors, receptor ligands, kinases, phosphatases, proteases are represented by the different shapes located next to the protein labels. Increased and decreased RNA expression of the genes is represented by the red and blue solid circles, respectively, with the intensity of the color representing the degree of differential expression. The green, red, and grey arrows indicate the interactions being positive/activation, negative/inhibition and unspecified, respectively.

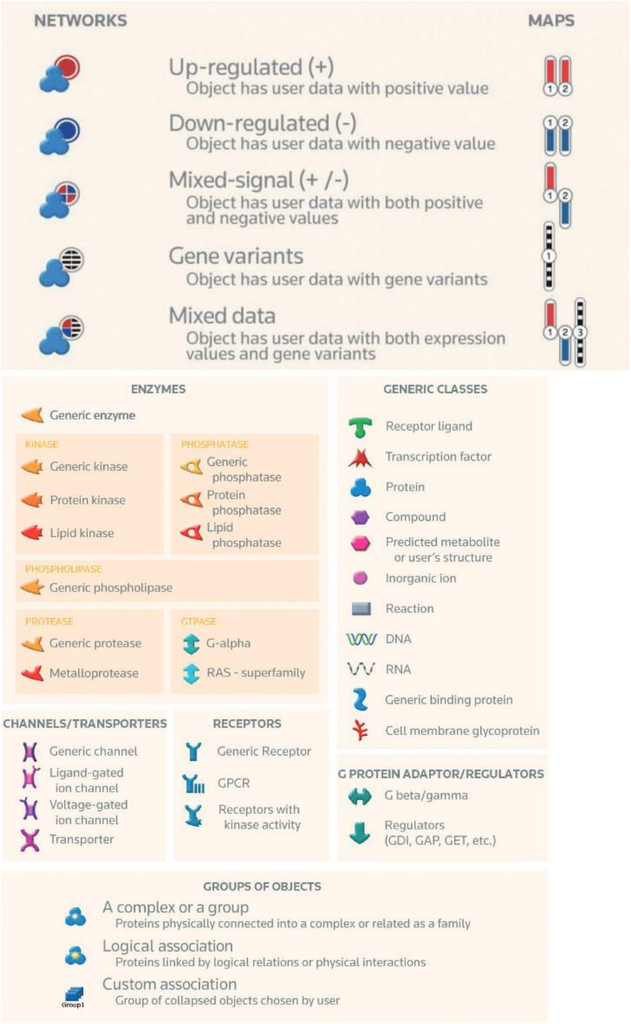

Supplement: Supplementary file 1 [file cells-11-01701-s001.zip › Supplementary Material S6_Figure Legend for the Metacore network.pdf]
